# A multimodal user interface for touchless control of robotic ultrasound

**DOI:** 10.1007/s11548-022-02810-0

**Published:** 2022-12-24

**Authors:** Josefine Schreiter, Tonia Mielke, Danny Schott, Maximilian Thormann, Jazan Omari, Maciej Pech, Christian Hansen

**Affiliations:** 1 Faculty of Computer Science and Research Campus STIMULATE, University of Magdeburg, Magdeburg, Germany; 2 University Hospital of Magdeburg, University of Magdeburg, Magdeburg, Germany

**Keywords:** Human–robot interaction, Multimodal interface, Robotic ultrasound, Touchless interaction

## Abstract

**Purpose:**

Past research contained the investigation and development of robotic ultrasound. In this context, interfaces which allow for interaction with the robotic system are of paramount importance. Few researchers have addressed the issue of developing non-tactile interaction approaches, although they could be beneficial for maintaining sterility during medical procedures. Interaction could be supported by multimodality, which has the potential to enable intuitive and natural interaction. To assess the feasibility of multimodal interaction for non-tactile control of a co-located robotic ultrasound system, a novel human–robot interaction concept was developed.

**Methods:**

The medical use case of needle-based interventions under hybrid computed tomography and ultrasound imaging was analyzed by interviewing four radiologists. From the resulting workflow, interaction tasks were derived which include human–robot interaction. Based on this, characteristics of a multimodal, touchless human–robot interface were elaborated, suitable interaction modalities were identified, and a corresponding interface was developed, which was thereafter evaluated in a user study with eight participants.

**Results:**

The implemented interface includes voice commands, combined with hand gesture control for discrete control and navigation interaction of the robotic US probe, respectively. The interaction concept was evaluated by the users in the form of a quantitative questionnaire with a average usability. Qualitative analysis of interview results revealed user satisfaction with the implemented interaction methods and potential improvements to the system.

**Conclusion:**

A multimodal, touchless interaction concept for a robotic US for the use case of needle-based procedures in interventional radiology was developed, incorporating combined voice and hand gesture control. Future steps will include the integration of a solution for the missing haptic feedback and the evaluation of its clinical suitability.

**Supplementary Information:**

The online version contains supplementary material available at 10.1007/s11548-022-02810-0.

## Introduction

Robotic ultrasound (US) has been studied several times in the past and has been developed for multiple medical domains [1]. The guidance of the US probe by the robot can be autonomous or under manual control of the user. However, even with autonomous robot control, it may be necessary to manually correct the position and orientation of the US probe. This requires an interface that enables communication with the robot. Touchless interaction techniques are conceivable and advantageous, as they help to maintain sterility in the medical environment. Moreover, the combination of multiple modalities mimicking human interaction could make the interaction with a robot more natural and intuitive [2]. The objective of this work is the development of a user-friendly interface for the touchless control of a robotic US for needle-based interventions. Therefore, a multimodal interface was developed based on a use case analysis and evaluated regarding its usability under laboratory conditions.

## Related work

### Robotic ultrasound

One part of research on assistive robots focuses on the development of robotic US [1]. These systems connect a US station to a robot, with the US probe being attached to the end effector of the robot. Robotic US can help to overcome the physical effort required during conventional US-guided procedures due to the simultaneous positioning of the needle and US probe and the required application of contact force [3]. In most developments, tactile interaction methods are used in the form of teleoperation [4, 5]. Advanced systems include haptic devices which enable force control [6, 7]. Systems with higher robot autonomy include, for example, automatic application of a US probe’s contact force while operators control the remaining degrees of freedom (DOF) of the US probe [8]. To date, there is no developed robotic US that proposes a multimodal interface for HRI.

### Multimodal human–robot interaction

Since human-to-human communication is inherently multimodal, combining multiple communication channels is used in HRI to enable natural and user-friendly interfaces. Generally, most research on multimodal HRI interfaces incorporates a combination of voice input and hand gestures, with the former used for discrete control of robots and the latter for continuous control. In preliminary work, the pose of the robot end effector was specified by hand gestures and the action was confirmed by voice input [9]. Perzanowski et al. [10] implemented a multimodal HRI using voice commands, hand gestures and tactile input via a handheld device, where two input modalities were each combined to evoke a robot behavior. The combinations are redundant to each other, so that the user can individually select a combination according to preferences. Wagner et al. [11] compared the multimodal input of voice commands and hand gestures to a combined voice and head gesture input. The application in a pick-and-place trial revealed that interaction via head orientation is more precise, faster and also perceived as less physically and mentally demanding. Other work involved augmented reality (AR) to provide interfaces to the user and/or to give visual feedback [12, 13].

## Use case analysis

Expert discussions were held with four radiologists experienced in needle-based interventions to define the procedure and requirements for use. The discussions focused on the workflow of current needle-based interventions under hybrid computed tomography (CT) and US imaging and requirements of a potential robotic US to be used during these procedures. Semistructured interviews were conducted with one radiologist at a time. For analysis, audio recordings were made and duplicate statements were clustered. The workflow described below is derived from the repeated statements of the radiologists. To develop a suitable interface for the HRI, the interaction tasks resulting from the workflow are identified.

### Workflow

Needle-based interventions under hybrid CT/US imaging are particularly useful for puncturing lesions in the kidney and liver. Intra-interventional imaging serves to track the needle within the body. Radiologists’ statements suggest that CT imaging is applied for initial puncturing before switching to US, as deeper risk structures such as blood vessels can be more easily detected under US. Unlike CT imaging, where only axial image slices can be acquired, US imaging is not tied to anatomic orientation, so the US probe can be oriented based on the needle path. The focus of the visualization is on the needle tip. However, it may also be necessary to visualize the target structure or risk structures located on the path. After successful positioning of the needle within the target anatomy, the needle position is verified by post-operative CT images.

### Interaction tasks

**Fig. 1:**
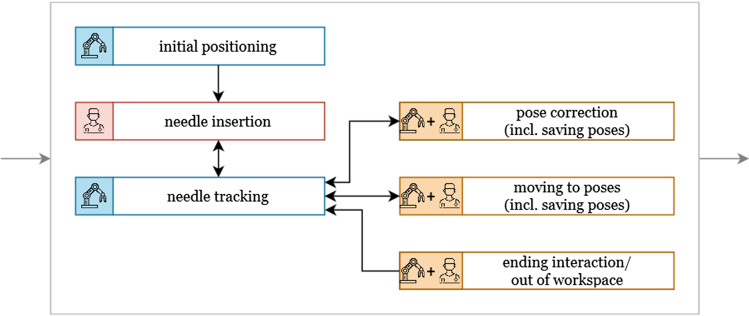
Tasks during needle-based interventions under hybrid CT/US imaging which are completed autonomously by the robot (blue), by the surgeon (red) or interactively (orange)

By using a robotic US, a partially automated workflow can be achieved (see Fig. 1). The initial positioning of the US probe based on the needle trajectory could be realized automatically, as well as the tracking of the probe when manually inserting the needle.

Due to errors in automatic tracking or obscuring of anatomical structures, it may be necessary to manually correct the pose of the US probe. This includes manual correction of the translational position and rotational orientation of the probe. There may also be a need to visualize anatomical structures located on the further needle trajectory that are not visible on the current US image. This requires the navigation of the US probe to arbitrary poses. Besides the manual navigation, it should also be possible to save any arbitrary probe pose and to move to it later. Furthermore, it should be possible to move the robot arm out of the workspace by a manual command, for instance, to finish the procedure.

**Table 1:** Overview of the integrated commands for voice control of the robotic US

Voice command	Explanation
Translation	Starts the translational navigation mode
Rotation	Starts the rotational navigation mode
Stop	Deactivates the current selected navigation mode & the detection of symbolic activation gestures
Save	Opens the visualization for assignment options for saving poses
Cancel	Closes the visualization for pose assignment options
Move to	Opens the visualization for assignment options for moving to saved poses
Sphere, Pyramid, Cube	Selects the assignment for saving and for moving to poses
End	Ends the current interaction task, moves the robot to the specified starting position and resets the saved poses
Start	Activates the detection of symbolic activation gestures

## Interface conception

The identified interaction tasks which require manual user input can be classified as discrete interaction and navigation interaction. In this context, discrete interaction tasks include the activation and deactivation of modes (e.g., translational motion), saving arbitrary probe poses and moving to them. Interaction tasks for navigation comprise moving the robot, and thereby the US probe, to an arbitrary position and orientation in space, hence to control six DOF. Manually controlling a US probe to generate images requires successive changes in its pose. Therefore, to enable manipulation, input modalities that support continuous input are required.

In the following, modalities for the identified interaction tasks are explored and determined. After that, the realization and implementation of the modalities are explained in detail.

### Modalities

The mapping of input modalities to discrete interaction tasks and those of navigation is based on results from a preliminary user study. The aim of the study was to determine the most appropriate combination of interaction modalities for discrete and navigation interaction. For this purpose, different combinations of interaction modalities were evaluated regarding accuracy, task completion time, influence on concurrent tasks and subjective feedback on usability. The combinations included voice and gaze control for discrete interaction, as well as hand and head gesture input for navigation control. Sixteen subjects with technical backgrounds participated in the study. Regarding the discrete interaction, it was shown that the feedback on the users’ subjective experience was more positive for the voice than for the gaze control. For the navigation interaction, it was found that the interaction task could be completed significantly faster when using hand gestures compared to head gestures. Consequently, for the further development of the interaction concept, it was decided to use voice input for discrete control and hand gestures for navigation interaction.

#### Voice interaction

For voice recognition, the commercially available recognition system *Microsoft Speech API*^1^ is used. Input commands are in German. However, for better understanding, they are explained in English in the following. Command language was preferred to natural language input because it can be recognized more robustly [14] and allows for more intuitive interaction [15].

An overview of included voice commands is given in Table 1. A LED ring at the robot flange provides visual feedback (see Fig. 4). During active navigation mode, the green LED lights up. Further visual feedback is provided by buttons shown in AR. During navigation, these provide feedback about the active navigation mode. For saving or moving to saved points, the possible assignments named according to the corresponding geometric objects are shown.

#### Hand gesture interaction

**Fig. 2:**
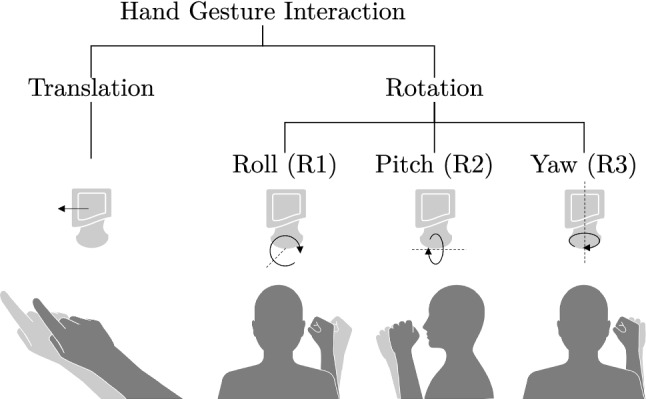
Overview of included hand gestures for continuous translational and rotational control of the robotic US. Rotational control is achieved by rotation of the shoulder joint (R1), flexion and extension of the elbow joint (R2) and rotation of the forearm (R3)

The control for translational and rotational DOF is separated because manipulation of all six DOF can cause incorrect inputs, such as unintentional rotation during translational control [14]. Because it is assumed that one hand might be needed for other tasks during the interventional workflow, such as needle insertion, a one-handed interaction is implemented. Translational motion control of the robot is achieved by moving the index finger in a pointing gesture (see Fig. 2). Controlling the rotation is performed by moving the forearm when it is upright (see Fig. 2).

Alternatively to voice interaction for discrete input, symbolic activation gestures of the hand for the purpose of starting and stopping the navigation modes are added. The symbolic activation gestures are held during the navigation interaction so that the execution of the gesture implicitly activates and deactivates the mode. A pinch gesture is used for translational control of the robot, and rotational DOF can be controlled by forming a fist. Activating the navigation modes using symbolic activation gestures is only possible in conjunction with the voice command *start*.

Translation of the robot is realized via relative hand movements by adding the direction vector to the current robot position. This vector is converted to robot coordinates based on the implemented registration transformation. The relative translation of the hand is transferred to robot motions in an unscaled manner. Direct transfer of the motions is limited by a maximum robot velocity and acceleration.

**Fig. 3:**
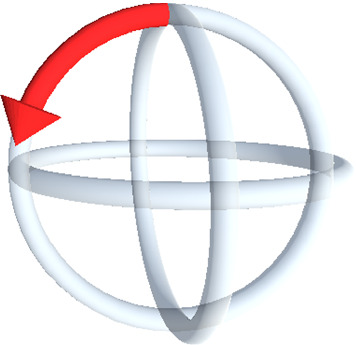
Visual feedback for rotational control of the robotic US given under AR

Changes in motion of the arm are mapped to the rotation of the end effector about discrete axes of rotation (see Fig. 2). Only changes in a rotation relative to the rest pose, above a defined threshold, result in a robot motion. The distance of the motion is determined by a transfer function. To prevent false interpretation of the rest pose, it is dynamically redefined each time the user returns to it. For the rotation control of the robot, visual feedback under AR is given to the user in the form of a widget (see Fig. 3). A hologram of it is displayed on the US probe. When an arm rotation changes above the defined threshold, the direction of the rotation resulting from the interaction is highlighted in the widget by a red arrow.

### System setup

**Fig. 4:**
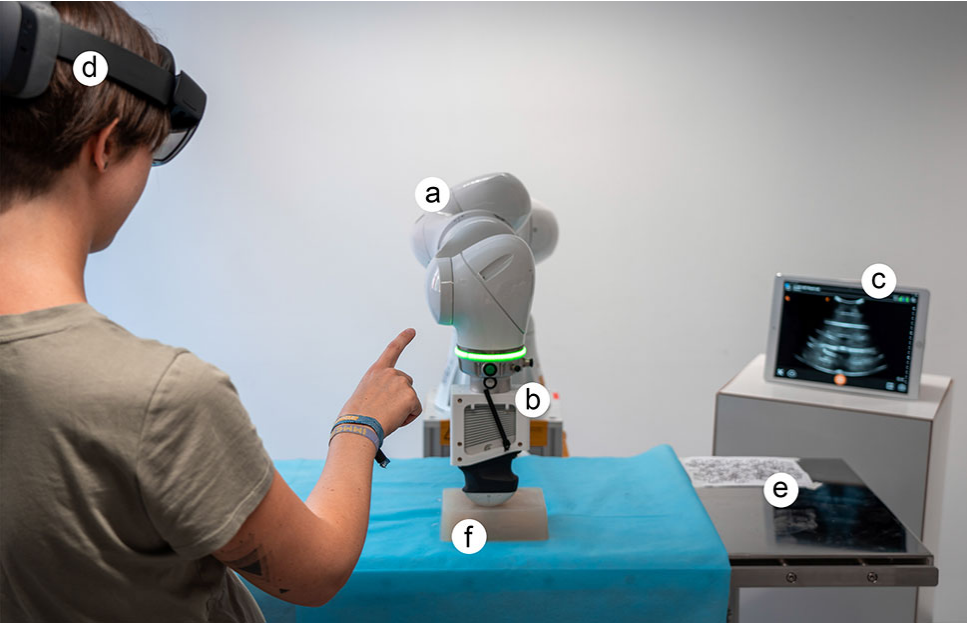
System setup of the robotic US during the user study including a robot arm (**a**), an attached US probe (**b**), a tablet showing live US images (**c**), an AR headset (**d**), an optical marker (**e**) and a custom-made phantom (**f**)

The robotic US encompasses a robot arm of type *KUKA lbr iiwa*^2^ (see Fig. 4a). A *Clarius US Scanner HD C3*^3^ with a custom-made adapter is attached to its end effector (see Fig. 4b). Live US images are sent over a local network to a workstation placed next to the robot (see Fig. 4c).

Since our System requires both input recognition and AR content display, a *Microsoft HoloLens 2* (see footnote 1) was chosen for prototype implementation (see Fig. 4d). It is implemented using the *Mixed Reality Toolkit* (see footnote 4) to incorporate hand and voice interaction and the *Vuforia SDK*^4^ to enable optical marker detection. To co-register the coordinate systems of the *HoloLens* and the robot, an optical marker is used (see Fig. 4e). Its coordinate system is localized when the application is started. *Unity*^5^ is used to implement the application.

The robotic motion control is realized with the help of *KUKA Sunrise.OS* (see footnote 2). The additional package *KUKA Sunrise.Servoing* (see footnote 2) facilitates continuous and high-frequency specifications of target points in real time. The motion commands sent always refer to the tool center point (TCP), which is located at the tip of the US probe. For safe HRI, a safety zone is defined. The robot’s blue LED ring is used to provide visual feedback to the user when approaching the boundaries of this zone with the TCP. In addition, the force in the z-direction is limited, meaning the pressure being applied with the US probe on surfaces. If the force exceeds a specified limit, the robot movement is stopped. In this case, the red LED provides visual feedback to the user.

The motion commands are sent from the *HoloLens* to the robot controller in byte-coded form via UDP connection. Because communication with the robot controller is only possible via Ethernet connection, the data are first sent to a desktop PC, which is connected to the *HoloLens* via a Wifi network.

## Evaluation

A user study under laboratory conditions was conducted to evaluate the usability of the designed interaction concept for the use case.

### Participants

The prerequisites for participation in the study were a medical background and experience in interpreting US images. Eight subjects (6 female, 2 male) between 24 and 29 years ($M = 26.6$, $\textrm{SD} = 2.0$) participated in the study. Of these, seven were medical students between the sixth and 15th semester ($M = 10.4$, $\textrm{SD} = 3.3$) and one was a medical resident. With regard to eyesight, five participants stated that they had no limitations and three stated that they had corrected eyesight. Participants rated their technical affinity on a 5-point Likert scale between two and five (1: low, 5: high, $M = 3.8$, $\textrm{SD} = 0.9$). Prior experience with touchless interaction techniques was estimated between two and five (1: no prior experience, 5: very experienced, $M = 2.9$, $\textrm{SD} = 1.1$) and with application of US imaging between three and five (1: no prior experience, 5: very experienced, $M = 3.4$, $\textrm{SD} = 0.7$).

### Apparatus

Two phantoms with identical components were developed for the study, one for training purposes and one for the conduction of the trial. Three differently shaped rubber objects were placed in each phantom (pyramid, cube, and sphere), as well as a rib-like structure made of plastic, which served to cause US attenuation, so that additional rotation of the US probe was necessary to visualize underlying objects. The placement of the objects differed in both phantoms. The phantom was placed on a table in front of the robot to guarantee its reachability with the US probe (see Fig. 4f).

### Measures

To get insights about the usability as an indicator of the feasibility of our interface, qualitative and quantitative measures were recorded using a think aloud protocol [16] with subsequent semi-structured interview and the system usability scale (SUS) [17], respectively. The latter questionnaire allows the collection of statistical data on perceived usability by answering 10 standardized questions on a Likert scale. During the interview, in addition to general questions, further questions were asked that are related to individual statements given during the trial. To analyze the statements made during the study, they were hierarchical clustered by combining statements that occurred at least twice into a summary statement. Subsequently, these statements were assigned to categories. In addition, the task completion time was recorded, defined as the time taken from the start of the interaction to the location of the last rubber object.

### Procedure

First, participants were asked to complete a questionnaire to collect demographic data. Second, an introduction to the topic was given, which included an explanation of the interaction techniques and the interaction task. Third, two training tasks were conducted. The first task aimed at learning to identify the target objects in US images. For this purpose, all objects within the training phantom had to be located consecutively by manually controlling the US probe without the use of the robot. The second training task aimed at learning the HRI methods. Several target poses of the US probe were given as holograms under AR. They had to be moved to using the HRI methods. In addition, the training included saving poses and moving to those. Third, if participants reported feeling confident in interpreting US images and in using the interaction methods, the study task was given. It consisted of locating the three objects existing in the US phantom in a constant predefined order. For this purpose, the integrated interaction techniques had to be used to position the robotic US in such a way that the respective target structure was visible on the US image. Saving and moving to saved US probe poses could be used to follow the given order. After all three objects were located in the requested order, the trial ended. Finally, participants were asked to complete the SUS questionnaire and the semi-structured interview was conducted.

## Results

The conduction of the study took approximately 45 min per participant, the task completion time was 544.84 s ($\textrm{SD} = 124.82$ s). The usability of the interaction concept was evaluated by the participants through the SUS questionnaire with a mean score of 69.9 ($\textrm{SD} = 18.0$). To classify the achieved result, it can be compared with the average score of 68 presented by Sauro [18]. Accordingly, the SUS score determined in the study can be rated as average.

Regarding the qualitative results of the study, 217 individual statements were recorded. Of these, 104 statements were mentioned by at least two participants. These were generalized into 38 statements and classified into 14 categories. These, in turn, were categorized into general feedback, feedback on discrete interaction and feedback on navigation interaction. The individual subcategories of the three feedback categories as well as the included generalized statements are shown in Table 2. The implemented interaction methods were considered by most participants as working well and intuitive, respectively. Moreover, drawbacks of the system could be identified. These include the display of important information outside the user’s field of view when looking at the US monitor, such as the visual feedback of the LED ring, and the current probe pose. Furthermore, participants criticized the lack of haptic feedback, and thus the difficulty in estimating the applied surface pressure.

**Table 2:** Summary of qualitative results of the user study. The ID indicates the corresponding participant

Category	Generalized statement	ID
*General*		
Functionality	Reactions of the system were predictable	1, 2, 3
	Automatic stopping of the robot was very good	6, 8
Modality	HoloLens is comfortable to wear	3, 7
	It takes some time getting used to HoloLens	6, 7
Phantom	Fixed points of orientation are missing	4, 5, 6
Feedback	LED ring is helpful	3, 4, 8
	LED ring is out of field of view when looking at monitor	3, 5, 6, 7
	Lack of haptic feedback	1, 2, 8
	Difficult to estimate the applied pressure	1, 4, 5
Additional functions	More accurate feedback on applied pressure	1, 6, 8
	Additional feedback if applied pressure too high	3, 5, 6, 8
	Additional support for orientation	3, 6, 8
*Discrete interaction*		
Voice command	Voice recognition worked well	3, 4, 5, 7
	Voice commands are intuitive	1, 3, 7
	*Move To* command is not intuitive	1, 3
	Voice interaction takes too long to select navigation modes	3, 5
Saving and moving to	Saving and moving to poses worked well	1, 2, 3
	Saving and moving to poses is practical	3, 5
Activation gestures	Activation gestures are too similar	2, 3, 5
	Alternative interaction to activate navigation modes would be helpful	3, 5
Additional functions	Command to perform large rotations	5, 6
	Commands to operate US functions	5, 7, 8
*Navigation interaction*		
General	Gestures for interaction are intuitive	1, 2, 4, 6, 7, 8
	Interaction can be learned quickly	2, 7
	Robot motions are comprehensible	4, 8
	Robot motions are very precise	4, 8
	Very fast transfer of gestures to robot motions	6, 7, 8
	Current probe pose difficult to assess when looking at monitor	2, 3, 4, 8
Translation	Motion on one level difficult	1, 2, 7
Rotation	Rotation challenging due to anatomical limitations of arm	3, 4, 5
	Practice needed to learn that start orientation of the arm can be adjusted for rotation	3, 4, 8
	Helpful that during rotation the position is kept constant	5, 8
Interaction space	Necessary to consciously remember field of view of the camera	1,3
	Camera field of view for gesture recognition too small	3, 4, 5
	Physically demanding that hand must be held up due to the interaction space	3, 4
	Hand position would be more stable if the hand did not have to be held freely	1, 8
Additional functions	Possibility to lock axes to allow motions on one level	2, 7
	Automatic maintenance of constant surface pressure	3, 7

## Discussion

This work extends previous work by evaluating the feasibility of touchless multimodal control of robotic US for user-friendly HRI. The proposed interface was rated by users with average usability. This is also reflected by the interview results, where although the interaction was described as intuitive, improvement potential emerged, which is discussed in the following.

During translational control, participants rated it difficult to move the US probe on the horizontal plane. This led to difficulties, especially when moving on the phantom surface, and affected imaging by loss of contact or resulted in unwanted, high contact forces on the phantom surface. The application of a pre-defined contact force after reaching the surface, like it was proposed in a work of Fang et al. [8], could address this problem. Moreover, participants evaluated it as challenging to perceive the given visual feedback (e.g., LED ring) and the US probe when looking at the US monitor. Hence, it is crucial to display all needed information in the user’s field of view. Therefore, it might be beneficial to unify the visualizations by, for example, displaying the feedback from the LED ring on the *HoloLens* or, as proposed in current research, displaying the ultrasound image in AR [19, 20]. Future work could also explore interfaces other than the *HoloLens* to address limitations such as interaction space and ergonomics. Moreover, the lack of haptic feedback during the control of the robotic US was criticized. To improve this, more precise information could be given about the applied contact force. One possibility would be displaying the applied contact force in the form of visual feedback, audio feedback [21] or, as mentioned before, the application of a pre-defined contact force [8].

It is plausible that a number of limitations have influenced the results obtained. One limitation arising from the study design is that no quantitative values, for example regarding latency or registration accuracy, were collected. Although errors in this regard may be negligible, as no comments were made in the qualitative feedback, these factors should be investigated in the future. Additionally the study considered an isolated task from the elaborated workflow requiring interactive control of the robot. Autonomous robot actions, such as needle tracking and the manual needle insertion by the radiologist, were disregarded. As a result, the significance of the findings obtained from the study is only partially valid with respect to the integrability of the developed interface into a realistic workflow. Furthermore, the phantom needs to be adapted in such a way that it depicts realistic human anatomies to enable orientation that is as close to reality as possible. This fact was also mentioned by participants during the interview as a limiting constraint encountered during the study. Another limitation results from the sample. The participants in the study indicated that they had moderate experience in the application of US. Higher experience levels of participants could have an influence on the orientation during imaging, as well as the handling of the missing haptic feedback. Consequently, in further studies, participants with different levels of experience should be recruited to obtain more comprehensive feedback on clinical suitability and possibilities for improvement. In addition, a larger number of participants should be included in further studies to potentially allow for better generalizations.

## Conclusion

This work involved the conception, development and evaluation of a touchless, multimodal user interface for interaction with a robotic US. Expert interviews were conducted to analyze the use case, which resulted in the identification of HRI tasks. The developed interface incorporates voice commands for discrete control, like mode selection and hand gestures, for the translational and rotational navigation of the robotic US probe. In a user study, the interaction concept was rated with an average usability. Results of the study moreover reveal future directions for improving the interaction concept. These include an integration of the missing haptic feedback and visualization of all needed information within the users’ field of view by, for example, displaying US images under AR. Therefore, this work has demonstrated that multimodal interaction has the potential to enable touchless, user-friendly control of robotic US, laying the foundation for future research on multimodal interaction for such systems in the medical field.

## Supplementary information

This work is accompanying a supplementary video to demonstrate the implemented multimodal, touchless interaction methods which includes navigational and rotational control of a robotic US.

## Supplementary Information

Below is the link to the electronic supplementary material.

Supplementary file 1 (mp4 43004 KB)

## Footnotes

1 Microsoft Corporation, USA.

2 KUKA AG, Germany.

3 Clarius Mobile Health, Canada.

4 PTC Inc., USA.

5 Unity Technologies, USA.

## References

[1] Von Haxthausen F, Böttger S, Wulff D, Hagenah J, García-Vázquez V, Ipsen S (2021). Medical robotics for ultrasound imaging: current systems and future trends. Curr Robotics Rep.

[2] Goodrich MA, Schultz AC (2007). Human–robot interaction: a survey. Found Trends® Hum Comput Interact.

[3] Craig M (1985). Sonography: an occupational health hazard?. J Diagn Med Sonogr.

[4] Huang Q, Lan J (2019). Remote control of a robotic prosthesis arm with six-degree-of-freedom for ultrasonic scanning and three-dimensional imaging. Biomed Signal Process Control.

[5] de Cunha D, Gravez P, Leroy C, Maillard E, Jouan J, Varley P, Jones M, Halliwell M, Hawkes D, Wells PNT, Angelini L (1998) The midstep system for ultrasound guided remote telesurgery. In: Proceedings of the 20th annual international conference of the IEEE engineering in medicine and biology society. Biomedical engineering towards the year 2000 and beyond (Cat. No. 98CH36286), vol 20. IEEE, pp 1266–1269

[6] Mathiassen K, Fjellin JE, Glette K, Hol PK, Elle OJ (2016) An ultrasound robotic system using the commercial robot ur5. Front Robotics AI 3:11

[7] Zandsteeg CJ, Bruijnen DJH, van de Molengraft MJG (2010) Haptic tele-operation system control design for the ultrasound task: a loop-shaping approach. Mechatronics 20(7):767-777

[8] Fang T-Y, Zhang HK, Finocchi R, Taylor RH, Boctor EM (2017). Force-assisted ultrasound imaging system through dual force sensing and admittance robot control. Int J Comput Assist Radiol Surg.

[9] Maurtua I, Fernández I, Tellaeche A, Kildal J, Susperregi L, Ibarguren A, Sierra B (2017). Natural multimodal communication for human–robot collaboration. Int J Adv Rob Syst.

[10] Perzanowski D, Schultz AC, Adams W, Marsh E, Bugajska M (2001). Building a multimodal human–robot interface. IEEE Intell Syst.

[11] Wagner P, Malisz Z, Kopp S (2014). Gesture and speech in interaction: an overview. Speech Commun.

[12] Hugle J, Lambrecht J, Kruger J (2017) An integrated approach for industrial robot control and programming combining haptic and non-haptic gestures. In: 2017 26th IEEE international symposium on robot and human interactive communication (RO-MAN). IEEE, pp 851–857

[13] Park K-B, Choi SH, Lee JY, Ghasemi Y, Mohammed M, Jeong H (2021). Hands-free human–robot interaction using multimodal gestures and deep learning in wearable mixed reality. IEEE Access.

[14] Preim B, Dachselt R (2015). Interaktive Systeme.

[15] Ferre M, Macias-Guarasa J, Aracil R, Barrientos A (1998) voice command generation for teleoperated robot systems

[16] van Someren M, Barnard YF, Sandberg J (1994). The think aloud method: a practical approach to modelling cognitive.

[17] Brooke J (1996). SUS: A ‘quick and dirty’ usability scale. Usability evaluation in industry.

[18] Sauro J (2011) A practical guide to the system usability scale: background, benchmarks & best practices. Measuring Usability LLC

[19] Nguyen T, Plishker W, Matisoff A, Sharma K, Shekhar R (2022). HoloUs: augmented reality visualization of live ultrasound images using HoloLens for ultrasound-guided procedures. Int J Comput Assist Radiol Surg.

[20] Rüger C, Feufel MA, Moosburner S, Özbek C, Pratschke J, Sauer IM (2020). Ultrasound in augmented reality: a mixed-methods evaluation of head-mounted displays in image-guided interventions. Int J Comput Assist Radiol Surg.

[21] Li T, Meng X, Tavakoli M (2022) Dual mode *p*HRI-*tele*HRI control system with a hybrid admittance-force controller for ultrasound imaging. Sensors (Basel, Switzerland) 22(11) 10.3390/s22114025 PMC9185235 35684646

